# Emotional valence and contextual affordances flexibly shape approach-avoidance movements

**DOI:** 10.3389/fpsyg.2013.00933

**Published:** 2013-12-13

**Authors:** Ana Carolina Saraiva, Friederike Schüür, Sven Bestmann

**Affiliations:** ^1^Sobell Department of Motor Neuroscience and Movement Disorders, UCL Institute of Neurology, University College LondonLondon, UK; ^2^Department of Psychology, New York UniversityNew York, NY, USA

**Keywords:** flexion, extension, pre-defined, affordances, fight-or-flight, common-coding, reference-frame

## Abstract

Behavior is influenced by the emotional content—or valence—of stimuli in our environment. Positive stimuli facilitate approach, whereas negative stimuli facilitate defensive actions such as avoidance (flight) and attack (fight). Facilitation of approach or avoidance movements may also be influenced by whether it is the self that moves relative to a stimulus (self-reference) or the stimulus that moves relative to the self (object-reference), adding flexibility and context-dependence to behavior. Alternatively, facilitation of approach avoidance movements may happen in a pre-defined and muscle-specific way, whereby arm flexion is faster to approach positive (e.g., flexing the arm brings a stimulus closer) and arm extension faster to avoid negative stimuli (e.g., extending the arm moves the stimulus away). While this allows for relatively fast responses, it may compromise the flexibility offered by contextual influences. Here we asked under which conditions approach-avoidance actions are influenced by contextual factors (i.e., reference-frame). We manipulated the reference-frame in which actions occurred by asking participants to move a symbolic manikin (representing the self) toward or away from a positive or negative stimulus, and move a stimulus toward or away from the manikin. We also controlled for the type of movements used to approach or avoid in each reference. We show that the reference-frame influences approach-avoidance actions to emotional stimuli, but additionally we find muscle-specificity for negative stimuli in self-reference contexts. We speculate this muscle-specificity may be a fast and adaptive response to threatening stimuli. Our results confirm that approach-avoidance behavior is flexible and reference-frame dependent, but can be muscle-specific depending on the context and valence of the stimulus. Reference-frame and stimulus-evaluation are key factors in guiding approach-avoidance behavior toward emotional stimuli in our environment.

## Introduction

Selecting appropriate actions in response to emotional stimuli is crucial for successful social and emotional interaction. For example, while it seems safe to approach and sit next to a smiling stranger on the bus, one should avoid a potentially dangerous scene of disturbance on the street. Indeed, emotions are thought to be fundamental predispositions for action (James, 1884; Frijda, 1986; Damasio, 1994): positive stimuli facilitate approach, whereas negative stimuli facilitate avoidance (e.g., withdrawal and escape) (Bradley et al., 2001; Lang and Bradley, 2010).

Approach and avoidance behavior may not be only shaped by the valence of the stimulus, but also by the context in which behavior occurs (common-coding account; Eder and Rothermund, 2008; Eder and Klauer, 2009). More specifically, behavior may be influenced by whether the environment requires us to move ourselves toward or away from an emotional stimulus (self-reference) or whether it requires us to move the emotional stimulus toward or away from ourselves (object-reference). Moreover, both arm extension *and* flexion can be used to approach or avoid: we can *extend* our arm to pet a puppy, or alternatively *flex* our arm to bring the puppy closer to us. Similarly, we can *flex* our arm to move away from a spider but also *extend* our arm to chip that spider away from us. Thus, to achieve the same behavioral goal, actions conducted in a self-reference frame are opposite to the ones conducted in an object-reference frame. This suggests that the reference-frame may determine which actions are coded as “approach” and “avoidance.” In the self-reference frame, for example, arm flexion may be coded as an avoidance action and would subsequently be facilitated upon *negative* stimulus evaluation. In the object-reference frame, by contrast, arm flexion may be coded as an approach action and, following stimulus evaluation, would be facilitated by a *positive* stimulus. Viewed in this way, the reference-frame may determine the “affordance of an action” (Gibson, 1977): flexion can elicit avoidance in a self-reference frame, whereas in an object-reference frame the same movement allows for approach.

An alternative suggestion is that *specific* movements may automatically be facilitated to approach or avoid emotional stimuli upon (conscious or non-conscious) stimulus evaluation (Chen and Bargh, 1999; Duckworth et al., 2002). We here refer to this suggestion as the “muscle-specific” account. Accordingly, we should be faster at *flexing* our arm to bring a positive stimulus closer to us (e.g., a puppy) and faster at *extending* our arm to push away a negative stimulus (e.g., a spider; Cacioppo et al., 1993; Chen and Bargh, 1999) independently of the context. Such automaticity would suggest a close but rigid relationship between emotional stimuli, selective muscle recruitment, and approach-avoidance behavior, which may be a hard-wired process that bypasses conscious awareness of the encountered stimuli (Lang and Bradley, 2010). This may allow for faster responses, but compromises the flexibility offered by contextual influences. To understand how contextual factors may allow for flexible actions therefore requires controlling for the presence of muscle-specificity within each reference-frame.

Evidence for the common-coding account has been substantial (Rotteveel and Phaf, 2004; Markman and Brendl, 2005; Lavender and Hommel, 2007; Bamford and Ward, 2008; Eder and Rothermund, 2008; Seibt et al., 2008; Proctor and Zhang, 2010; see Table 1). To demonstrate movement flexibility, studies have varied either the reference-frame (Lavender and Hommel, 2007; Seibt et al., 2008) or the arm movements required to approach and avoid (e.g., flexion vs. extension to approach; Markman and Brendl, 2005; Proctor and Zhang, 2010), but never both. Similarly, other work supporting the common-coding account suggested that when the self approaches and avoids, behavior is a goal-dependent mechanism rather than muscle-specific (Bamford and Ward, 2008), but in this work there was no manipulation of neither the reference-frame nor movement type. Manipulation of both factors, however, is required to test for movement flexibility within each reference-frame. Studies in support of the muscle-specific account (Duckworth et al., 2002; Roelofs et al., 2009) did not manipulate the reference-frame or movement type either (for an overview of studies on approach and avoidance and the experimental factors included in their designs, see Table 1) and could not reveal muscle-specificity in different contexts.

**Table 1:** **Overview of studies supporting the muscle-specific vs. common-coding accounts**.

**Paper**	**Manipulation type**		
	**Reference-frame**	**Movement type (flex/ext)**	**Valence RT**
Chen and Bargh, 1999	No—Object-reference only	No—Flexion to approach, extension to avoid	No—averages RTs across valences
Markman and Brendl, 2005	No—Object-reference only	Yes—Both flexion and extension movements were made for approaching and avoiding	Yes
Lavender and Hommel, 2007	No—Self-reference only	No—Flexion to approach, extension to avoid	No
Eder and Rothermund, 2008	No	Yes—Both flexion and extension movements made for approaching and avoiding—not directly compared within the same experiment	No
Seibt et al., 2008	Yes—Both reference-frames	No—Flexion to approach, extension to avoid in the object-reference; extension to approach and flexion to avoid in the self-reference	Yes
Bamford and Ward, 2008	Yes—Self-reference only	No—Extension to approach and avoid	Yes
Van Dantzig et al., 2009	No—Object-reference only	Yes—Both flexion and extension movements were made for approaching and avoiding	Yes
Krieglmeyer et al., 2010	No—Self-reference only	No—No flexion or extension was executed	No
Proctor and Zhang, 2010	No—Object-reference only	Yes—Both flexion and extension movements were made for approaching and avoiding	Yes
Current experiment	Yes—Both reference-frames	Yes—Both flexion and extension movements were made for approaching and avoiding	Yes

The table summarizes the experimental factors included in previous approach-avoidance studies. We included studies that depict situations in which the self moves toward or away from a stimulus, or a stimulus is moved toward or away from the self, irrespective of the requirement to move a symbolic self/object on a screen. **Reference-frame**: Was a manipulation of reference-frame included? In the self-reference frame, the self always moves toward or away from an emotional stimulus. In the object-reference frame, an emotional object is always moved toward or away from the self. **Movement type**: Were flexion movements made to approach and avoid, and extension movements made to approach and avoid emotional stimuli, or was a pre-defined set of movements used? **Valence influences on RT**: Was the relative influence of positive and negative valences on RTs assessed?

To investigate the extent of flexible movement facilitation for approach-avoidance within each reference-frame, one needs to demonstrate that within each reference-frame there is facilitation of a variety of movements and that one type of movement (e.g., flexion) is not *per se* faster than another (e.g., extension) when approaching and avoiding stimuli. Studies showing that approach and avoidance of emotional stimuli is influenced by the reference-frame, for example, only included specific subsets of flexion and extension movements to approach or avoid emotional stimuli. In Lavender and Hommel (2007), subjects always made an extension when the self moved toward (approached) and always made a flexion when the self moved away from (avoided) emotional stimuli. Similarly, in Seibt et al. (2008), in the self-reference frame condition subjects pushed (extended) a joystick to move toward and pulled (flexed) to move away from a stimulus. Essentially, both studies assumed that specific arm movements are naturally facilitated in self-reference situations, namely arm extension when the self approaches, and flexion when the self avoids. No direct comparison was made with flexion movements for approach and extension movements for avoidance within a self-reference frame, which is essential to eliminate any potential confounds with the biophysical properties of the arm.

The direct comparison of movements to approach and avoid emotional stimuli has only been conducted in an object-reference frame, in which both flexion and extension movements were facilitated when moving a stimulus closer to and away from a reference on the screen (Markman and Brendl, 2005; Van Dantzig et al., 2009; Proctor and Zhang, 2010). It remains undetermined whether this flexible facilitation of flexion and extension movements extends to the self-reference frame (i.e., when the *self* moves toward or away from stimuli). Directly comparing different reference-frames allows one to distinguish how different interchangeable contexts affect approach-avoidance. This crucial manipulation is essential to reveal the extent of movement flexibility within different contexts.

In contrast to previous studies (Chen and Bargh, 1999; Lavender and Hommel, 2007; Van Dantzig et al., 2008; Krieglmeyer et al., 2010), we looked at participants' reaction times for positive and negative stimuli separately. Approach-avoidance behavior occurs in response to the valence of the stimuli in our environment. That is, approach is typically linked to positive and avoidance is typically linked to negative valences. However, negative stimuli are known to facilitate various defensive actions such as escape or attack (Lang and Bradley, 2010), which are not necessarily linked to avoidance (i.e., moving *away* from the stimulus). Thus, given that negative stimuli can trigger a variety of defensive actions, it may be possible that negative stimuli do not only facilitate avoidance. To demonstrate the how approach and avoidance is influenced by emotional stimuli, we looked at reaction times for both valences separately. We also administered questionnaires that would help us determine any subjective influences on our reaction times. Factors such as personality and motivational traits may influence the subjective interpretation of various emotional stimuli, and consequently determine approach and avoidance behavior (Elliot and Thrash, 2010).

No study, to the best of our knowledge, has directly compared the effects of different movement types within different reference-frames on approach-avoidance behavior (see Table 1; current experiment). Combining these three factors within the same experimental design is key for understanding the individual contributions of reference-frame, movement type and valence to approach and avoidance behavior, and, crucially any interactions between them. The manipulation of reference-frame allows for observing contextual influences on approach-avoidance. Similarly, the manipulation of movement type controls for the potential presence of muscle-specificity within different reference-frames. And the manipulation of valence allows us to demonstrate the individual contribution of each valence to approach-avoidance. Our design therefore allows us to uncover interactions other work has not been able to show, for example, muscle specificity within one reference-frame for one type of valence only; only full factorial designs can uncover interaction effects.

We used a design that incorporated two different arm movements (flexion/extension) used to approach or avoid, varied the reference-frame in which these movements occurred, and balanced the valence of the emotional stimuli that had to be approached or avoided. To this end, we devised a novel paradigm which combined two tasks commonly used to study approach-avoidance behavior: the manikin task (De Houwer et al., 2001) and the joystick task (Chen and Bargh, 1999). This combination improves previous versions of the manikin task (De Houwer et al., 2001; Krieglmeyer et al., 2010) in that the movement of the symbolic manikin on the screen is actually linked to arm flexion and extension, which is crucial for our purposes. This allows participants to see the behavioral consequences of their actions, as either a decrease or increase in the distance between the self and the emotional stimulus. Furthermore, it controls for the possibility that apparent movement facilitation can be due to the specific biophysical constraints of different arm movements: some movements may be more difficult to execute than others. Finally, we manipulate reference-frame by moving the symbolic manikin toward or away from a stimulus or a stimulus toward or away from the manikin. This design allowed us to manipulate the reference-frame (self vs. object) and movement type (extension vs. flexion) *independently.*

If the common-coding account holds true, reference-frame should flexibly influence facilitation of approach-avoidance movements (i.e., the consequences of stimulus evaluation determines whether we approach or avoid) *and* the specific movements that are facilitated (i.e., both flexion and extension can be used to approach and avoidance within the particular context). That is, the common-coding account predicts an interaction of the reference-frame, stimulus valence and approach-avoidance actions. In contrast, the muscle-specific account predicts an interaction of movement type with stimulus valence (i.e., specific actions will be activated by emotional stimuli to achieve faster approach or avoidance). That is, participants should pull faster to approach positive and push faster to avoid negative stimuli, irrespective of the reference-frame. We therefore predicted possible interaction effects occurring between the reference-frame, movement type and valence, which previous work had not been able to reveal.

In brief, we show that the reference-frame influences which actions are facilitated to approach and avoid, and also determine the speed at which we approach and avoid emotional stimuli, in line with previous findings. We found no evidence of muscle-specificity in object-reference conditions. In contrast, in the self-reference we found evidence of movement flexibility for positive stimuli and muscle-specificity for negative stimuli. We provide novel, albeit speculative, evidence that negative (threatening) stimuli have the capacity of triggering a variety of defensive actions in a fast and efficient manner. We conclude that flexible approach-avoidance behavior is dependent on the reference-frame.

## Methods

### Participants

28 healthy volunteers (mean age = 25.7 ± 5.95 SD years; 17 female) were recruited from the University College London Psychology database, with local ethics approval (UCL). All participants had normal or corrected-to-normal vision. Subjects gave written informed consent to participate in the study, and received monetary compensation for their time and travel (10 £/h). Two participants were excluded from the analysis for having a total number of errors above 2 SD's from the population mean for self and object references. Picture ratings from one subject were missing and the subject was excluded from the questionnaire correlation analysis. Overall, 26 subjects were included in the main analysis and 25 subjects were used in the picture ratings analysis and questionnaire correlations analysis.

### Stimuli and apparatus

220 pictures were selected from the International Affective Picture System (IAPS; Lang et al., 2008): 110 negative pictures (e.g., gang violence, mutilated bodies) with valences 1.8–3.8 (1.8 = most negative), and 110 positive pictures (e.g., babies, families) with valences 6.2–8.2 (8.2 = most positive). All pictures (maximum 240 × 180 pixels) were matched for luminance. Pictures differed in their arousal ratings, which we addressed in our analyses. We included pictures depicting only humans or animals as we believed those to represent what most humans interact with on a daily basis. The assignment of pictures in each of the 8 blocks and order of presentation was randomized for each subject. Block order was randomized per subject such that no two subjects had the same sequence of block instructions or same sequence of pictures in each block. Each block comprised of 110 trials (55 positive/negative). In total, for each reference-frame, there were 110 repetitions for each experimental condition.

All stimuli were presented on an 19 inch LCD monitor of a Dell Optiplex-780 computer (refresh rate: 60 Hz), using the Cogent 2000 toolbox (University College London, http://www.vislab.ucl.ac.uk/Cogent2000/index.html) and MATLAB (v.7.9.1.705; The MathWorks Inc.). A white manikin (2.8 × 1.9 cm) represented the self. A small yellow circle (1.3 × 1.2 cm) representing the “away” target was presented at the edge of the screen on the same side as manikin presentation. This “away” target was presented purely for guidance purposes when subjects had to move away. The manikin or picture stimuli were moved smoothly on the screen using a home-made analog joystick connected to a CED1401 (Cambridge Electronic Design) machine. Joystick data were recorded using a CED1401-MATLAB interface (matced32; Cambridge Electronic Design) between MATLAB and the data acquisition device.

### Experimental procedure and design

One experimental session was conducted for each reference-frame, in a counter-balanced order, and each session was separated by at least 1 day. At the start of the experiment, participants completed 3 questionnaires: the Big Five Personality Inventory (BFI; John et al., 1991), the Behavioral Activation and Inhibition Scales (BIS/BAS; Carver and White, 1994) and the Fear Survey Schedule [FSS-III; (Wolpe and Lang, 1964)]. Participants then sat in front of a computer at a distance of approximately 50 cm with the joystick fixed to the table on the right-hand side. They were instructed to imagine that the manikin represented themselves on the screen.

**Figure 1:**
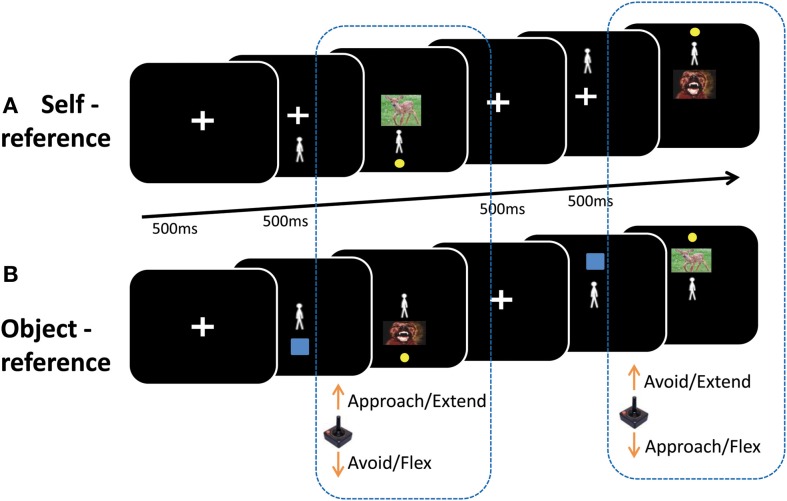
**Illustration of the task. (A)** Self-reference condition: a manikin was presented either above or below an emotional picture. Participants moved the manikin toward or away from the picture depending on the block. In *congruent* blocks (approach-positive, avoid-negative) participants were instructed to move the manikin with a joystick toward positive pictures and away from negative pictures, as fast as possible upon picture presentation. In *incongruent* blocks (approach-negative, avoid-positive) participants had to move toward negative and away from positive pictures. **(B)** Object-reference condition: an emotional picture was presented either above or below a manikin. Participants moved the emotional picture toward or away from the manikin depending on the block. Participants were instructed to move positive pictures toward and negative pictures away from the manikin (*congruent* blocks), or move negative pictures toward and positive pictures away from the manikin (*incongruent* blocks). For both reference-frames, the direction of movement determined whether subjects had to make a pull or a push to approach and avoid.

In the *self-reference* condition, participants moved a manikin (the self) on the screen toward or away from an emotional picture. Previous work has shown that participants can represent themselves on a computer screen when moving positive or negative stimuli toward or away from their own names (Markman and Brendl, 2005). Here, we represent the subject on the screen by means of a symbolic manikin, which has produced reliable approach-avoidance effects (De Houwer et al., 2001; Krieglmeyer and Deutsch, 2010; Krieglmeyer et al., 2010). Trials started with a fixation cross presented in the center of the screen (500 ms), followed by both the fixation cross and the manikin (500 ms), which was presented pseudo-randomly either above or below fixation (see Figure 1A). Central fixation was then replaced by a picture (max. 2000 ms, or until the respective target was reached). The inter-trial-interval was 500 ms. Within this condition, two types of blocks occurred, each repeated four times. In *congruent* blocks (approach-positive, avoid-negative) participants were instructed to move the manikin toward positive pictures and away from negative pictures, as fast and as accurately as possible upon picture presentation. In *incongruent* blocks (approach-negative, avoid-positive) participants had to move toward negative and away from positive pictures. The starting position of the manikin (above or below) determined the type of movement that was executed to approach and avoid. When the manikin was located above, subjects had to pull to approach and push to avoid. When the manikin was located below, subjects had to push to approach and pull to avoid. Therefore, both approach and avoidance could be achieved by either pulling or pushing, depending on the starting position of the manikin.

In the *object-reference* condition, participants moved an emotional picture (object) toward or away from a centrally presented manikin. Trials started with the central fixation cross (500 ms), followed by both the fixation and a blue square that was pseudo-randomly presented above or below fixation (500 ms; see Figure 1B). Subsequently, the central fixation was replaced by the manikin and the blue square was replaced by the picture (max. 2000 ms, or until the target was reached). Participants were instructed to move positive pictures toward and negative pictures away from the manikin (*congruent* blocks), or move negative pictures toward and positive pictures away from the manikin (*incongruent* blocks). Here, the starting position of the picture also determined the type of movement that subjects had to execute to approach or avoid.

*Congruency* thus refers to whether the goal of approaching or avoiding was congruent with the valence (i.e., approach positive and avoid negative pictures). *Movement type* refers to whether a pull or a push was executed to approach and avoid the emotional stimulus.

For *each* reference-frame session, participants completed two training blocks of 30 trials each, one for congruent and one for incongruent movements, before the main experiment to familiarize themselves with the task. For training, a different set of 60 pictures (30 negative/positive) was used. Participants rated the valence of all the pictures at the end of the second experimental session on a scale of 1–9 (1 = most negative; 9 = most positive). They did not provide arousal ratings.

### Data analyses

We measured reaction times (RT; time from picture onset to joystick movement onset) and movement times (MT; time from movement onset to completion of the movement, i.e., reaching the target area) as dependent variables. Joystick data were recorded in XY-coordinates and RTs were calculated based on vertical y-axis coordinate deviations (over 10 coordinates) from trial baseline. The baseline was recorded while the manikin remained stationary prior to the appearance of the picture. All trials in which the movement did not match the instructions (e.g., avoiding when instructed to approach negative images; 10.2% of trials), with RTs below 100 ms (0.6%) or exceeding 2000 ms (3.8%) were excluded from analyses. Outlier RTs (2.5%) were calculated per block for each subject using Grubb's test (α = 0.05) and were excluded. Across both experimental sessions, 17.1% of trials were excluded.

We conducted a 2 × 2 × 2 × 2 within-subjects repeated measures ANOVA (RM-ANOVA) with factors *Reference-Frame* (self vs. object) × *Movement type* (pull vs. push) × *Congruency* (congruent vs. incongruent) × *Valence* (positive vs. negative) for both RT and MT analyses. For all analyses, we report partial η^2^ as a measure of effect size. For all significant results, statistical threshold was fixed at 0.05. Significant interactions were followed-up using two-tailed paired-sample *t*-tests. Mean RTs are reported in brackets, ±1 SD. For questionnaire correlations, we used a 2-tailed bivariate Pearson correlation and corrected for multiple comparisons.

To analyse the effect of arousal on RTs, we conducted a multiple regression for each condition with dependent variable RT and independent variables *valence* and *arousal* for each participant separately. We then computed a 4-Way RM-ANOVA on the beta coefficients obtained from the multiple regression with factors *Reference-Frame* (self vs. object) × *Variable type* (valence vs. arousal) × *Movement direction* (approach vs. avoid) × *Movement type* (pull vs. push). The beta coefficients were taken as an indication of which of the independent variables (valence or arousal) had a greater impact on the RTs.

## Results

### Reaction times

**Figure 2:**
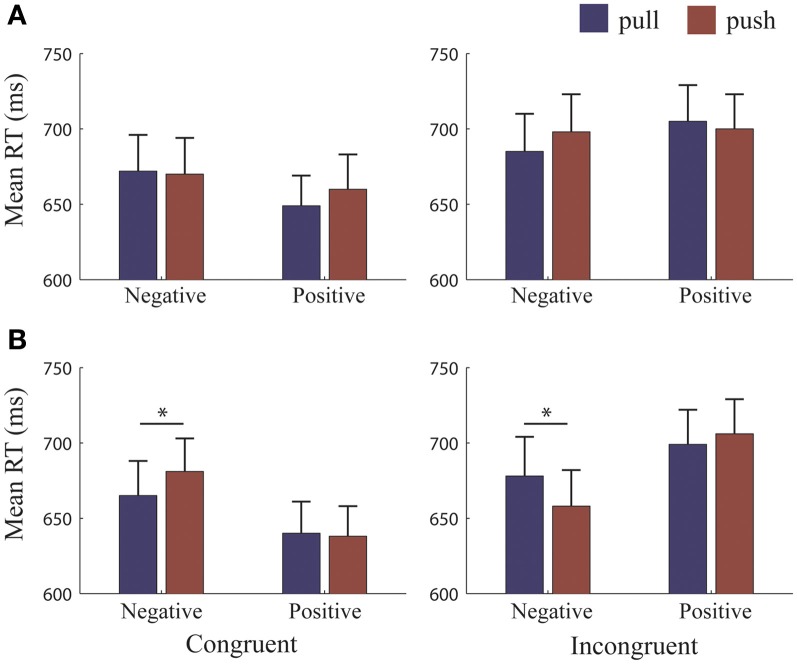
**RTs for each reference-condition**. Mean reaction times (RTs) for each reference-frame. **(A)** Object-reference condition: RTs did not differ when pulling or pushing to approach and avoid. **(B)** Self-reference condition: RTs were significantly faster for pulling to avoid and pushing to approach negative pictures. Error bars (+1 SE); blue bars, pull; brown bars, push. ^*^, significant at >= 0.001 level.

The 4-Way RM-ANOVA yielded a 4-way interaction across all four factor types [Ref.-Frame^*^Congruency^*^Valence^*^Movement type, *F*_(1, 25)_ = 33.31, *p* < 0.001, η^2^_*p*_ = 0.57]. All other interactions were qualified by this 4-way interaction, and we therefore only report follow-up statistics for the breakdown of this 4-way interaction. We split the 4-way interaction and analyzed separately for each *Reference-Frame* (Figure 2).

**Figure 3:**
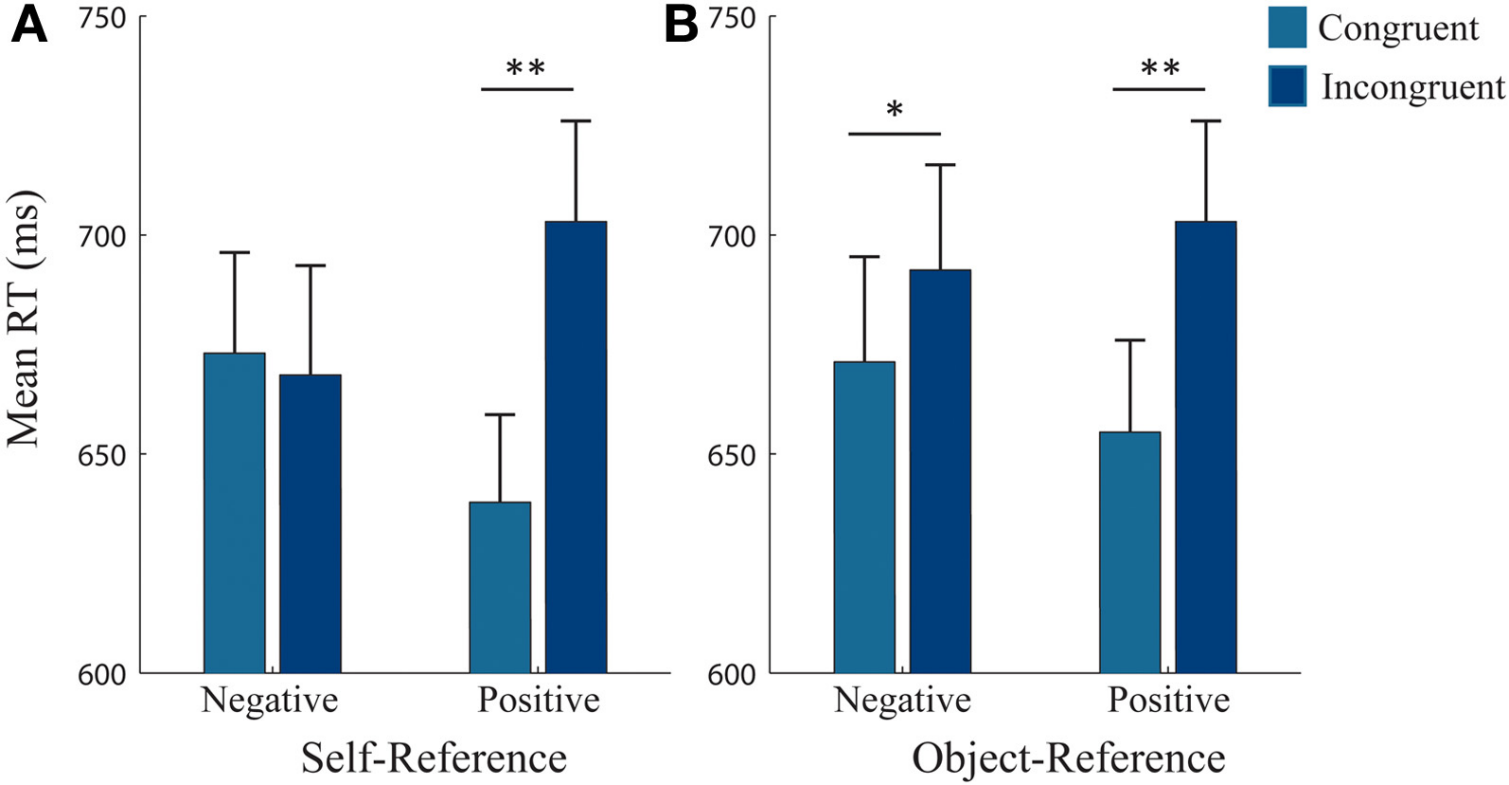
**RTs for each reference condition, collapsed across movement type**. Mean reaction times (RTs) for each reference-frame collapsed across pull and push movements. **(A)** Self-reference condition: RTs were significantly faster when participants approached compared to avoided positive pictures. **(B)** Object-reference condition: RTs were significantly faster for approaching than avoiding positive pictures and marginally significantly faster when avoiding compared to approaching negative pictures. Error bars (+1 SE); light blue bars, congruent; dark blue bars, incongruent. ^**^, significant at <0.001 level; ^*^, marginally significant (*p* = 0.087).

Follow-up analyses for the *object-reference* yielded a 3-way interaction [*F*_(1, 25)_ = 5.62, *p* = 0.026, η^2^_*p*_ = 0.18]. Paired *t*-tests did not reveal any significant differences between making push and pull movements when avoiding and approaching positive and negative pictures (Figure 2A). Thus, this 3-way interaction was mainly driven by a Congruency^*^Valence interaction [*F*_(1, 25)_ = 10.09, *p* = 0.004, η^2^_*p*_ = 0.29] which revealed a congruency effect for positive and negative valence, regardless of the type of movement made to achieve the required behavior (Figure 3B). Reaction times (RTs) were faster when approaching (655 ± 108 ms) compared to avoiding (703 ± 119 ms) positive pictures [*t*_(25)_ = −5.18, *p* < 0.001], and were marginally faster at avoiding (671 ± 122 ms) compared to approaching (692 ± 125 ms) negative pictures [*t*_(25)_ = −1.78, *p* = 0.087].

Follow-up analyses for the *self-reference* (Figure 2B) also yielded a 3-way interaction [*F*_(1, 25)_ = 52.00, *p* < 0.001, η^2^_*p*_ = 0.42]. In contrast to the *object-reference*, paired *t*-tests revealed differences between push and pull movements but only for negative pictures. We found that RTs were faster when subjects had to avoid negative pictures by pulling (665 ± 23 ms) than pushing (681 ± 22 ms) [*t*_(25)_ = −2.26, *p* = 0.032]. Similarly, RTs were faster when subjects had to approach negative pictures by pushing (658 ± 23 ms) than pulling (678 ± 26 ms) [*t*_(25)_ = 3.61, *p* = 0.001]. No other significant differences were found. Therefore, this interaction was mainly driven by differences between the valences, but also by differences between pushing and pulling for negative pictures only.

For the *self-reference* there was also a significant 2-way interaction between Congruency^*^Valence, [*F*_(1, 25)_ = 61.79, *p* < 0.001, η^2^_*p*_ = 0.71] which showed a congruency effect for positive pictures (Figure 3A). RTs were faster when approaching (639 ± 101 ms) compared to avoiding (703 ± 116 ms) positive stimuli, irrespective of the required action [*t*_(25)_ = −4.81, *p* < 0.001]. However, we did not observe a congruency effect for negative pictures (Avoid: 673 ± 115 ms, Approach: 668 ± 125 ms, n.s.). Thus, participants were equally fast in approaching or avoiding negative pictures, independently of the type of movement that was required.

### Movement times

The time it took for the initiated movement to be completed (i.e., MT) was also influenced by the reference-frame and the type of movement made. Analysis of the MTs revealed a 4-way interaction [Ref.-Frame^*^Congruency^*^Valence^*^Movement type, *F*_(1, 25)_ = 10.76, *p* = 0.003, η^2^_*p*_ = 0.30]. We analyzed this interaction separately for each *Reference-Frame*.

**Figure 4:**
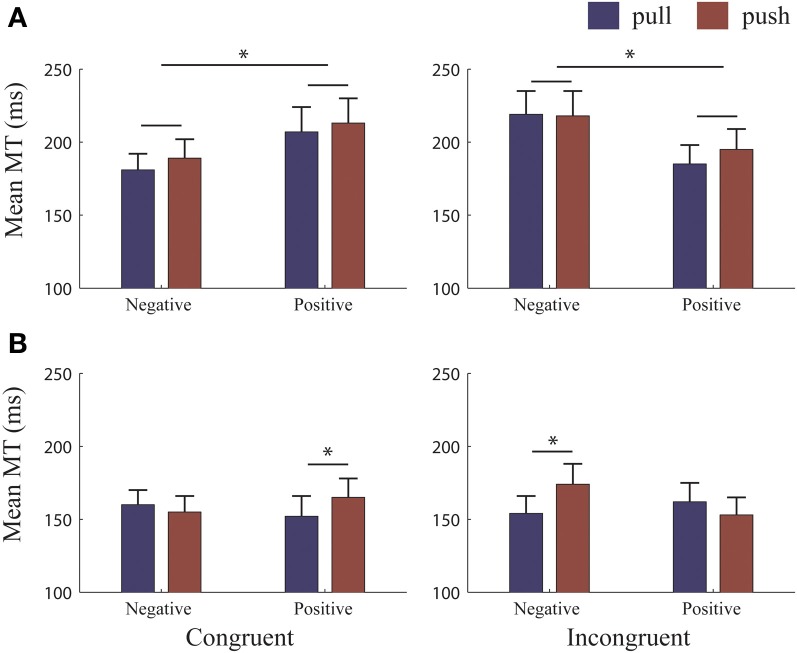
**MTs for each reference condition**. Mean movement times (MTs) for each reference-frame. **(A)** Object-reference condition: MTs did not differ when pulling or pushing to approach and avoid albeit MT to avoid were faster than to approach both valences. **(B)** Self-reference condition: MTs were significantly faster for pulling to approach both positive and negative pictures. There were no significant differences in MT to approach or avoid both valences. Error bars (+1 SE); blue bars, pull; brown bars, push. ^*^, significant at >0.001 level.

In the *object-reference* (Figure 4A), the 3-Way RM-ANOVA (with factors *Congruency, Valence and Movement type*) revealed a 2-way interaction Congruency^*^Valence [*F*_(1, 25)_ = 12.12, *p* = 0.002, η^2^_*p*_ = 0.33]. MTs were faster when moving to avoid (185 ± 59 ms) compared to moving to approach (219 ± 81 ms) negative pictures [*t*_(25)_ = −3.84, *p* = 0.001]. Similarly, MTs for avoiding positive pictures (190 ± 68 ms) were faster than approaching (210 ± 81 ms), [*t*_(25)_ = 2.08, *p* = 0.048]. Importantly, these effects occurred independently of the type of movement made. Thus, subjects were not only faster to initiate avoidance compared to approach when negative pictures were presented (as seen in the RTs) but they were also faster to move the picture away once the movement had been initiated. However, the result for positive pictures is difficult to interpret since RTs were faster for approach compared to avoidance, but subjects were faster at moving a positive picture away than toward.

In the *self-reference* (Figure 4B), follow-up analyses revealed a 3-way interaction [Congruency^*^Valence^*^Movement type, *F*_(1, 25)_ = 23.10, *p* < 0.000, η^2^_*p*_ = 0.48]. We split this interaction into each *Congruency*. For *Congruent* trials, pulling to approach positive pictures was faster than pushing [pull: 152 ± 14 ms; push: 165 ± 13 ms; *t*_(25)_ = −2.44, *p* = 0.022]. Differences between pushing and pulling to avoid negative pictures were not significant. For *Incongruent* trials, pulling to approach negative pictures was faster than pushing [pull: 154 ± 12 ms; push: 174 ± 14 ms; *t*_(25)_ = −3.30, *p* = 0.003]. Differences between pushing and pulling to avoid positive pictures were not significant. Thus, in situations where participants had to approach valenced stimuli, pulling was always faster than pushing. However, these differences for pulling or pushing movements to approach were the same for both valences, as revealed by paired sample *t*-tests. In contrast, when participants had to avoid both valences, there were no differences between movements. This highlights that without appropriate control, MT can potentially be explained by the biophysical properties of the arm rather than by the content of the stimulus. In sum, valence did not affect the time it took to execute the movement when the self was approaching or avoiding (see also Rotteveel and Phaf, 2004).

### Individual picture ratings and arousal

Subjects may differ in their perception of emotional stimuli, which may affect individual RTs. For this reason, we re-analyzed the data using participants' subjective picture ratings instead of the IAPS ratings. A 4-Way RM-ANOVA identical to that computed for the main RT analysis above revealed the same 3-way interaction [Ref.-Frame*Congruency*Valence, *F*_(1, 24)_ = 28.89, *p* < 0.001, η^2^_*p*_ = 0.55]. Follow-up ANOVA's yielded the same results as the main analysis above. Therefore, results were no different from the IAPS ratings.

Similarly, because emotional pictures also elicit arousal (i.e., intensity of activation; Lang and Bradley, 2010) we examined whether RT effects could be explained by arousal rather than valence. Arousal ratings were taken from the IAPS. Average arousal rating for positive pictures was 5 and for negative pictures 5.7. The 4-Way RM-ANOVA revealed an effect of the variable type on approach and avoidance [2-way interaction Variable type^*^Movement direction, *F*_(1, 25)_ = 10.80, *p* = 0.003, η^2^_*p*_ = 0.30]. We expected a positive correlation between valence and avoidance and a negative correlation between valence and approach. Indeed, we found that beta coefficients were significantly higher in the valence-avoidance (0.13 ± 0.03) compared to the valence-approach (−0.09 ± 0.06) conditions [*t*_(25)_ = 3.002, *p* = 0.006]. Arousal-avoidance was not significantly higher than arousal-approach. Thus, the arousal associated with the emotional stimuli did not influence our results.

### Questionnaire correlations

Some research has suggested that approach-avoidance tendencies may be influenced by motivation and personality traits (Elliot, 2006; Elliot and Thrash, 2010). For this reason, we tested whether personality traits, motivational states, and fear levels correlate with participants' individual picture ratings and RTs for approach-avoidance. Positive questionnaire items (BAS *drive*, *fun seeking*, *reward responsiveness*; BFI factor *Extraversion*) were correlated with ratings and RTs for positive pictures. Positive picture ratings and *Extraversion* correlated with each other (*r* = 0.54, *n* = 25, *p* = 0.005), indicating that more extroverted individuals rate stimuli more positively compared to those with low extraversion. Thus, more optimism is related to increased positive evaluation of emotional stimuli, compared to participants with a more pessimistic outlook. No other questionnaire ratings correlated with RTs for approaching positive stimuli. This may be because extraversion relates to affective experience (e.g., being outgoing or sociable), whereas approach is related to a broader range of biologically relevant emotional stimuli. Similarly, no correlations were found for BAS items because they are mainly elicited by reward associated social situations (Elliot and Thrash, 2010). Negative questionnaire items (*BIS*; BFI factor *Neuroticism*; FSS-III (*fear*)) items were correlated with ratings and RTs for negative pictures. There was a correlation trend between *Neuroticism* and *Fear*, (*r* = 0.46, *n* = 25, *p* = 0.021), but was not significant after correcting for multiple comparisons. No other correlations were observed. Overall, personality traits, social motivational states and fear levels did not impact on approach-avoidance behavior.

## Discussion

Approach-avoidance actions to emotional stimuli may be flexibly influenced by the context in which behavior occurs. Here, we tested for the extent of such flexible context-dependent behavior by comparing two different reference-frames and by manipulating the type of movements executed within each reference-frame.

### Reference-frame facilitates different actions for approach and avoidance

We show that the reference-frame influences the facilitation of flexion and extension movements to approach or avoid emotional stimuli. When the self had to move toward or away from a positive stimulus (self-reference), our results were incompatible with a muscle-specific account: when approaching positive pictures, pulling was not faster than pushing, and vice versa. Thus, we show support for the common-coding account and confirm previous studies (Rotteveel and Phaf, 2004; Markman and Brendl, 2005; Lavender and Hommel, 2007; Bamford and Ward, 2008; Eder and Rothermund, 2008; Seibt et al., 2008; Proctor and Zhang, 2010).

In contrast, we found novel evidence of muscle-specificity for negative pictures. When the self avoided negative pictures, pulling was faster than pushing, and when the self approached negative pictures, pushing was faster than pulling. Thus, defensive behavior facilitated specific movements when the self moved either toward or away from a negative stimulus. This supports previous work that push movements are faster when the self approaches a stimulus (e.g., a puppy), whereas pulling is faster for avoidance of a stimulus (e.g., a spider) (Wentura et al., 2000; Lavender and Hommel, 2007; Seibt et al., 2008). Importantly, this effect is opposite to that proposed by the muscle-specific account, namely that flexion is faster for approach and extension is faster for avoidance. It is therefore the context that determines which actions are facilitated. The muscle-specificity of approaching and avoiding negative pictures supports the existence of a fast-acting and automatic response mechanism to threat. Responding quicker to negative stimuli in our environment when the self needs to withdraw from a stimulus may have evolutionary advantages, and we speculate that this muscle-specificity arises from a hard-wired, possibly subcortical, process (LeDoux, 2000).

In the object-reference condition, the facilitation of actions to approach and avoid positive and negative stimuli was not pre-defined and muscle-specific. That is, when participants were required to move a stimulus toward or away from the self, there were no differences in initiating (RTs) and making (MTs) approach or avoidance movements. Thus, contrary to the predictions of the muscle-specific account, pulling was not faster than pushing to approach, and pushing was not faster than pulling to avoid. Our results replicate and confirm those of Markman and Brendl (2005) who had previously manipulated movement direction in an object-reference frame and demonstrated the actions to approach and avoid were not muscle-specific. Rotteveel and Phaf (2004) had also previously shown that although approach and avoidance were faster for positive and negative faces, respectively, such behavior was not automatically dependent on flexion or extension movements. Similarly, Eder and Rothermund (2008) suggested that it is not the pull or push action made that drives approach-avoidance behavior but rather the instruction that is assigned to the responses.

Our results therefore advocate the flexibility of pull and push actions to approach and avoid in both reference-frames, but also demonstrate a degree of muscle-specificity present only in situations when the self has to respond to threat. Thus, we confirm that actions to approach and avoid are not rigidly determined, as previously speculated (Rotteveel and Phaf, 2004; Bamford and Ward, 2008; Eder and Rothermund, 2008; Seibt et al., 2008). That is, when we want to chip a spider away extension is not faster than flexion and we want to pet a puppy or when we want to bring a puppy closer to us, extension is not faster than flexion. However, we also show that in some instances of threat, muscle-specific actions can be facilitated, supporting the existence of a fast-acting defensive circuitry. Importantly, our effects could not be attributed to arousal or personality traits, confirming that approach and avoidance are valence-driven behavioral effects.

### Reference-frame determines the affective coding of approach-avoidance actions

In the current experiment, we show that approach and avoidance behavior is influenced by whether it is the self that moves relative to an object or whether it is an object that is moved relative to the self. Thus, context determines how to approach or avoid emotional stimuli. While in the self-reference condition participants were faster to approach than avoid positive pictures, there was no difference between approaching and avoiding negative pictures (see also, Gawronski et al., 2005; Stins et al., 2011). In contrast, in the object-reference condition, participants were also faster to approach than avoid positive stimuli but avoidance was now faster than approach for negative pictures. Whereas, the object-reference showed the typical avoidance pattern for negative stimuli, the self-reference did not. These differences indicate that, through our experimental set up, subjects viewed both reference-frames as being different and therefore both tasks could be used interchangeably. Previous studies had shown that movements made to approach and avoid can occur to a salient reference on the screen (Van Dantzig et al., 2009; Proctor and Zhang, 2010). In line with previous work (cf Markman and Brendl, 2005), our results show that a symbolic self on the screen is capable of eliciting different approach-avoidance results for different reference-frames. We show that directly looking at interchangeable approach-avoidance contexts is important to determine context-dependent approach-avoidance effects.

It has been previously shown that negative stimuli can trigger a variety of defensive actions including escape (i.e., avoidance) and attack (i.e., approach) (LeDoux, 1999; Lang and Bradley, 2010). The current findings reinforce this hypothesis by showing that the self can facilitate movements either toward or away from a negative picture with equal speed. This tendency may result from the fact that our set of pictures contained both fearful and violent scenarios, which have been classified as being the most threatening from a survival perspective (Bradley et al., 2001). We *speculate* that the former may predominantly facilitate “flight” responses (i.e., the self moving away), whereas the latter may trigger “fight” responses (i.e., the self moving toward), which may be coded as appropriate responses to negative stimuli. Previous work had suggested that approach-avoidance is faster when there is a correspondence between the affective content and associated approach-avoidance response codes (e.g., positive-move toward, negative-move away; Proctor and Zhang, 2010). However, we find no evidence of such correspondence for negative stimuli, as moving toward was also coded as a negative response.

We speculate that the specific content of the negative stimulus (e.g., fearful vs. attack) may determine which behavior is facilitated (approach or avoid) and in self-reference contexts, the evaluation of negative stimuli may not necessarily result in avoidance. Indeed, recent work using whole-body movements suggests that the body approaches negative (attack) stimuli faster than it avoids such stimuli (Naugle et al., 2011). Similarly, faster avoidance has been linked to fearful faces (Seidel et al., 2010), and faster approach to angry faces (Carver and Harmon-Jones, 2009; Wilkowski and Meier, 2010; though see Stins et al., 2011). Furthermore, in the self-reference frame there was no difference in approach or avoidance speed once the movement was initiated (MTs). This reinforces the crucial role of stimulus evaluation in dictating differences in approach and avoidance, rather than any subsequent perceptual or cognitive mechanisms that may take place once the movement has been initiated, supporting studies that have shown an “evaluative-dependency” of approach-avoidance behavior (Rotteveel and Phaf, 2004; Lavender and Hommel, 2007; Eder and Rothermund, 2008). However, this result is at odds with previous work which has suggested that negative stimuli automatically trigger avoidance, both with and without conscious evaluation of the stimulus (Chen and Bargh, 1999; Rotteveel and Phaf, 2004; Krieglmeyer et al., 2010).

Crucially, in the self-reference frame, pulling (flexing) was faster to avoid and pushing (extending) was faster to approach negative stimuli, consistent with previous suggestions (Wentura et al., 2000; Lavender and Hommel, 2007). Thus, we may not only have a tendency to approach and avoid negative pictures depending on their level of threat, but for each tendency, specific movements are facilitated in a fast and efficient manner, suggesting a fast-acting context-dependent defensive circuitry (LeDoux, 2000).

Lastly, our results highlight the importance of looking at approach and avoidance effects for positive and negative stimuli separately. Thus, previously reported approach-avoidance effects (Chen and Bargh, 1999; Lavender and Hommel, 2007; Van Dantzig et al., 2008; Krieglmeyer et al., 2010) may mask important differences in reactions to valenced stimuli due to the practice of averaging RTs across positive and negative valences for congruent (i.e., approach-positive/avoid-negative) and incongruent (i.e., avoid-positive/approach-negative) conditions. Although previous work attempted to eliminate any confounding effect of stimulus evaluation on RTs by averaging across valences, our results highlight that a combination of stimulus evaluation and movement facilitation drive differences in RTs. Our work raises the interesting question whether avoidance effects can be further subdivided according to the specific requirement for flight or fight actions in response to negative stimuli. Given the current results, we suggest future studies directly address this by comparing approach and avoidance to pictures that have been classified as containing violent/attack scenarios and pictures depicting fearful stimuli.

### Alternative explanations

An alternative explanation suggests that approach and avoidance is dictated by the subjective outcome of the action (a decrease or an increase in the distance between the self and the emotional stimulus), rather than by stimulus evaluations (distance regulation account; Strack and Deutsch, 2004). The current design allowed participants to directly observe the outcome of their actions, and therefore show whether there was an actual increase or decrease in distance between the self and the stimulus. The results for negative pictures in the self-reference condition do not support the distance regulation account because in this condition the self both increased and decreased the distance This provides additional evidence for the evaluative nature of approach-avoidance, showing that the evaluation of the content of the picture dictates the facilitated action and that this occurs independently of the distance change involved.

## Conclusions

To conclude, we have shown that the reference-frame influences approach and avoidance, and flexibly determines the facilitation of actions to approach and avoid emotional stimuli. Thus, our data support the common-coding account by showing that action facilitation to approach and avoid emotional stimuli is not pre-determined and muscle-specific. Only when the self moves relative to a negative (threatening) stimulus, specific movements are triggered to either approach or avoid, similar to a “fight or flight” response, depending on whether stimulus evaluation elicits attack or escape. Our results therefore confirm that approach-avoidance behavior is flexible and reference-frame dependent, but can be muscle-specific depending on the context and valence of the stimulus. Our results demonstrate how reference-frame and stimulus-evaluation are key factors in guiding approach and avoidance behavior toward emotional stimuli in our environment.

### Conflict of interest statement

The authors declare that the research was conducted in the absence of any commercial or financial relationships that could be construed as a potential conflict of interest.
